# Encouraging Hearing Loss Prevention in Music Listeners Using Personalized Technology: Questionnaire Study

**DOI:** 10.2196/24903

**Published:** 2022-06-27

**Authors:** Dylan Tianyu Zhu

**Affiliations:** 1 Blair Academy Blairstown, NJ United States

**Keywords:** mHealth, mobile health, prevention, NIHL, noise induced hearing loss, MIHL, music induced hearing loss, intervention, wearable device

## Abstract

**Background:**

Noise-induced hearing loss (NIHL) affects millions of people despite being almost completely preventable. For recreational music listening through personal listening equipment (such as earbuds), it seems that listeners do not yet have a way to accurately assess their risk of developing hearing loss and prevent it accordingly.

**Objective:**

The aim of this study is to analyze the perceived utility of a hypothetical device that encourages NIHL prevention based on listeners’ exposure to noise and to determine the most effective methods of such encouragement. Here, we describe 3 different potential NIHL risk notification method types, as follows: auditory, external visual, and visual.

**Methods:**

An open, web-based survey was created on Google Forms, and the link was posted to Amazon’s Mechanical Turk as well as music-related Reddit communities. The survey was designed to gauge each respondent’s self-assessed NIHL awareness, willingness to lower their audio if reminded, and NIHL risk notification type preference. The likelihood of a specific notification type to encourage NIHL prevention among its users was based on the average of each user’s responses to 2 survey questions. Data collection started on July 13, 2020, and ended on July 17, 2020.

**Results:**

Of the 116 respondents, 92 (79.3%) reported having prior awareness about NIHL; however, 60 (51.7%) described doing nothing to prevent it despite 96 (82.8%) feeling a moderate, high, or extreme risk of developing NIHL. Of those who already prevented NIHL, 96% (53.5/56) described using estimates to guide their prevention instead of using data. A Kruskal-Wallis test corrected for ties showed that despite the visual NIHL risk notification type being selected by the highest number of participants (84/116, 72.4%), the auditory type had a significantly higher (H_1_=6.848; *P*=.03) average percentage likelihood of encouraging NIHL prevention (62%, SD 24%) among the 40 respondents who chose it, with a median likelihood of 56% (95% CI 50%-75%). The visual type’s average likelihood was 50% (SD 28.1%), with a median of 50% (95% CI 37.5%-56.3%). Regardless of the NIHL risk notification type, 69% (80/116) of respondents were not opposed to using NIHL risk notifications and lowering their audio volume accordingly.

**Conclusions:**

The hypothetical device detailed here was thought to be useful because most respondents (82.8%, 96/116) felt an extreme to moderate risk of developing NIHL and such a device could provide accurate data to those who currently use estimates to prevent NIHL, and most respondents were willing to act on NIHL risk notifications. The most effective NIHL risk notification type seemed to be the auditory type, but many aspects of this study need further research to determine which implementation method should reach the public.

## Introduction

### Background

Listening to music is a common recreational activity that occurs in a variety of settings. Although music is often played at a safe volume, if it is played at a sufficiently high volume for a long enough period, it can result in permanent noise-induced hearing loss (NIHL) [1]. An estimated 12.5% of children and adolescents aged 6-19 years (approximately 5.2 million in the United States) and 14% of adults aged 20-69 years (approximately 27.7 million in the United States) have NIHL [2,3]. From 1988 to 2010, Su and Chan [4] did not find a significant increase or decrease in the prevalence of NIHL in adolescents aged 12-19 years, whereas there was a consistently large percentage of the population with NIHL. Across that time frame, the incidence of NIHL in adolescents of that age seemed to be between a lower bound of 13% in the National Health and Nutrition Examination Survey 2009-2010 and an upper bound of 27% in National Health and Nutrition Examination Survey 2007-2008 [4]. Su and Chan [4] also mentioned that adolescent exposure to loud music through headphones increased whereas the use of hearing protection declined. As a nationally representative data set, estimates from these results indicate a large number of NIHL cases among adolescents in the United States. The already problematic incidence rate of NIHL is exacerbated by the fact that NIHL is both irreversible and largely preventable [5,6].

### Occupational and Recreational NIHL Differences

NIHL can arise from noise in both occupational and recreational settings [5,7]. Recreational hearing loss is sometimes referred to as music-induced hearing loss, a more specific version of NIHL. As both terms refer to hearing loss caused by noise, NIHL will be the term used in this study. With the rise of personal listening equipment and a general lack of education on NIHL prevention, NIHL is poised to remain a large public health issue in the future [4,8]. Although in the past NIHL was mainly considered to be caused by occupational noise, an increasing number of studies have started citing leisure time sounds as a significant contributor to the development of NIHL [6].

Recreational hearing loss is different from other types of NIHL, which can make it harder to combat. Whereas employers must adhere to regulations for acceptable occupational noise exposure set by the National Institute for Occupational Safety and Health (NIOSH) or the International Organization for Standardization, individual music listeners have complete freedom to adjust the noise entering their ear. If listeners are unaware of the safe noise exposure limits, they can easily exceed them [8]. NIOSH deems a permissible level of noise exposure as noise equal to 8 hours of an equivalent continuous sound level of 85 dB with an exchange rate of 3 dB [9]. Without the equipment to measure and calculate one’s incurred noise exposure, especially factoring in the 3-dB exchange rate, consumers can rely only on estimation. As Mercier and Hohmann [10] found that 60% of attendants at a music event did not perceive potentially dangerous audio levels (>87 dB) to be too loud and that 71% already suffered some degree of tinnitus, it seems likely that consumers do not already have the ability to effectively prevent NIHL on their own.

Another aspect of the problem is that a listener’s enjoyment of their music can be tied to their music’s volume [5]. Mercier and Hohmann [10] found that many young people believe that music is enhanced when played loudly. Kageyama [11] also found that among a group of 46 participants whose median age was 18 years, the sound levels they usually listened to were significantly higher than the levels at which they were comfortable hearing, suggesting that adolescents valued the volume of their music over their personal comfort and possibly the health of their ears. Thus, even if an individual is aware of the risks of NIHL, they may not have the incentive or necessary data to effectively prevent the development of NIHL [5]. Other studies indicate that even when individuals are aware of their risk of developing NIHL, they are still reluctant to use hearing protection [12,13].

### Proposed Solution

As there is strong evidence that NIHL can accumulate from a variety of sources [14], care should be taken to reduce NIHL from controllable sources. Preventing recreational hearing loss early is also paramount as it could limit the possibility of incurring greater total hearing loss over the course of one’s lifetime [14].

Once NIHL is obtained, the effects of NIHL could perennially cause great emotional, financial, and social stress that could otherwise be avoided [6]. Beyond the more apparent negative effects that NIHL can have on one’s quality of life, such as not being able to understand daily conversation, NIHL can also affect the income one receives [15,16]. Neitzel et al [17] found that *considerably conservative* estimates of the economic benefit of preventing NIHL could be anywhere from US $58 billion to US $152 billion in higher salaries.

Kaplan-Neeman et al [18] have previously shown the feasibility of using personal listening equipment and mobile apps to calculate and monitor listening habits. Their study found that people often inaccurately estimate their own listening habits. Participant self-reports for both listening levels and volume settings were only moderately correlated (*r*=0.655 and *r*=0.624, respectively) with the actual volume settings. Regardless, even a correct estimation of the actual volume setting would not necessarily mean that the participant had an awareness of their risk of developing NIHL. A total of 22% (8/37) of the participants surpassed the NIOSH daily noise exposure guideline at least once during the 14-day monitoring period.

### Existing Solutions

Unlike other potential contexts for music such as concerts, which already have products to mitigate NIHL risk (eg, earplugs), existing products for recreational music listening do not seem detailed enough to be useful to consumers. Apple does allow iPhone operating system (iOS; Apple Inc) 14 users to monitor the output decibel levels of their AirPods through what is called *control center*; however, the control center will not actively notify users about their listening without the user checking it first. In addition, the warning does not factor in the personal listening equipment’s noise output over a period, which is an important aspect of NIOSH’s regulation. The warning also does not seem to provide much more discrimination of risk to the user than simply always limiting a certain volume output.

There are many sound meter apps on both the Google Play and Apple App stores, but most only record the noise levels entering the personal listening equipment’s microphones or the microphones on the mobile devices themselves, as opposed to recording the output audio of the personal listening equipment itself. Different personal listening equipment may also output the same audio signal differently, which will need to be accounted for on a case-by-case basis. In addition, of the iOS apps we checked which measure the output audio of the earbud itself, many only support newer system versions such as Apple’s Health app for iOS 13+, which may limit the number of consumers that could easily make use of the product. A future review of such existing technologies will help better examine the utility of the device discussed in this paper.

### Study Objectives

This study seeks to build on the recommendation by Kaplan-Neeman et al [18] to create a technological solution for consumers to objectively monitor their listening habits and better encourage individual NIHL prevention. Providing listeners with some reminder about their music volume based on actual data (rather than estimation) can provide them with the information they need to be more cautious about their own chronic loud noise exposure, especially in response to the sensation-seeking tendency often associated with loud noise [11].

This study is not a randomized controlled trial, as no intervention was applied. It was only a survey. The survey questions were designed to gauge each respondent’s self-reported understanding of NIHL and to determine which hypothetical NIHL risk notification method respondents would respond most effectively to. In addition to a phone app NIHL risk notification similar to the method described by Kaplan-Neeman et al [18], 2 additional methods were considered here: auditory notifications (eg, a voice played through the user’s personal listening equipment) and external visual notifications (eg, some external device that is not a mobile phone, such as a watch). In the survey, the use case for each NIHL risk notification was described to respondents as being limited to situations where they were listening to music through personal listening equipment such as earbuds or headphones.

It was hypothesized that a significant majority of the population that uses personal listening equipment to listen to music is not aware of NIHL, do not have accurate methods to proactively analyze and prevent NIHL on their own, and are willing to reduce their music volume if reminded to do so. Thus, it was also hypothesized that technology that informs users of their risk of developing NIHL would be useful.

## Methods

### Survey Design

An open, web-based survey was created on Google Forms with 11 total questions, of which 3 were adaptive follow-up questions. The complete survey can be found in Multimedia Appendix 1. There were 5 pages of questions, of which 2 housed the adaptive follow-up questions (located on pages 2 and 4). The number of questionnaire items per page ranged from 5 to 1. Participants were able to return and change their answers before their survey submission. Before final form submission, they were not given a complete summary of their choices; however, they were able to request an automated summary of their answers after submission. The question order was not randomized among survey respondents. Only for a couple of questions were respondents able to provide a nonresponse answer, which took the form of a free response answer option. The data from the survey were automatically captured in a Google spreadsheet linked to the form. A statement was included at the beginning of the survey to ensure that respondents understood the privacy of the responses they provided, the purpose of the study, and all participants confirmed that they read the privacy statement and consented to having their responses used for this study. Participants were not told about how long or where the data would be stored, nor were they told who the investigator was. As this study was not connected to any institution, we did not apply for approval by an institutional review board.

After asking about the respondent’s age, the following three questions were aimed at understanding each survey taker’s familiarity with NIHL:

1. “Before this survey, had you heard of noise-induced hearing loss?”
2. “Do you believe you are at risk for developing noise-induced hearing loss?”
3. “Have you been diagnosed with noise-induced hearing loss?”

If a respondent answered “Yes” to the question, “Have you been diagnosed with NIHL?” they were then asked to describe the cause or causes of their NIHL. If a respondent answered “No,” they moved on to the next section. The following four questions sought to understand each participant’s willingness to mitigate their risk of NIHL:

1. “Do you do anything to actively prevent noise-induced hearing loss?”
2. “Would you like to receive some notification about your risk of developing noise-induced hearing loss from music or the environment around you?”
3. “Would you lower the volume of audio entering your ear if you were reminded?”
4. “Please select all of the notification methods that you would prefer to receive.”

If a respondent answered “Yes” to the question, “Do you do anything to actively prevent NIHL?” they were additionally asked if they used quantitative techniques to prevent NIHL. They were also asked to describe their NIHL prevention methods. The 2 questions that asked if the participant would like to receive NIHL risk notifications and if they would be willing to lower their audio volume both used the same 5-point Likert scale. The 5 answer choices on that scale spanned a range of qualitative responses, as follows: definitely, probably, maybe, probably not, and definitely not. The question about NIHL risk notification preferences was set up so that participants were able to select as many notification types as they preferred, meaning that respondents could each cast votes for more than one method. The available choices were audio, external visual, and visual notifications on your phone, as well as 2 additional choices, other and none. At the end of the survey, a final question was included simply asking respondents to write anything extra they might like to include. For workers on Amazon’s Mechanical Turk (MTurk), an additional question asked for their worker ID, and their survey completion screen also included a survey code which workers were asked to paste into MTurk to check for attentive survey completion. The survey was administered in two separate phases: the first phase was to collect responses from MTurk workers, and the second was for responses from Reddit users (Redditors).

### Collecting Data Using Amazon’s MTurk

MTurk is a website that allows people to be able to answer small tasks. The only requirement for workers on the site is to be aged ≥18 years. MTurk workers neither evidently share a common ideology nor are grouped in some easily discernible way compared with groups on Reddit.

**Figure 1:**
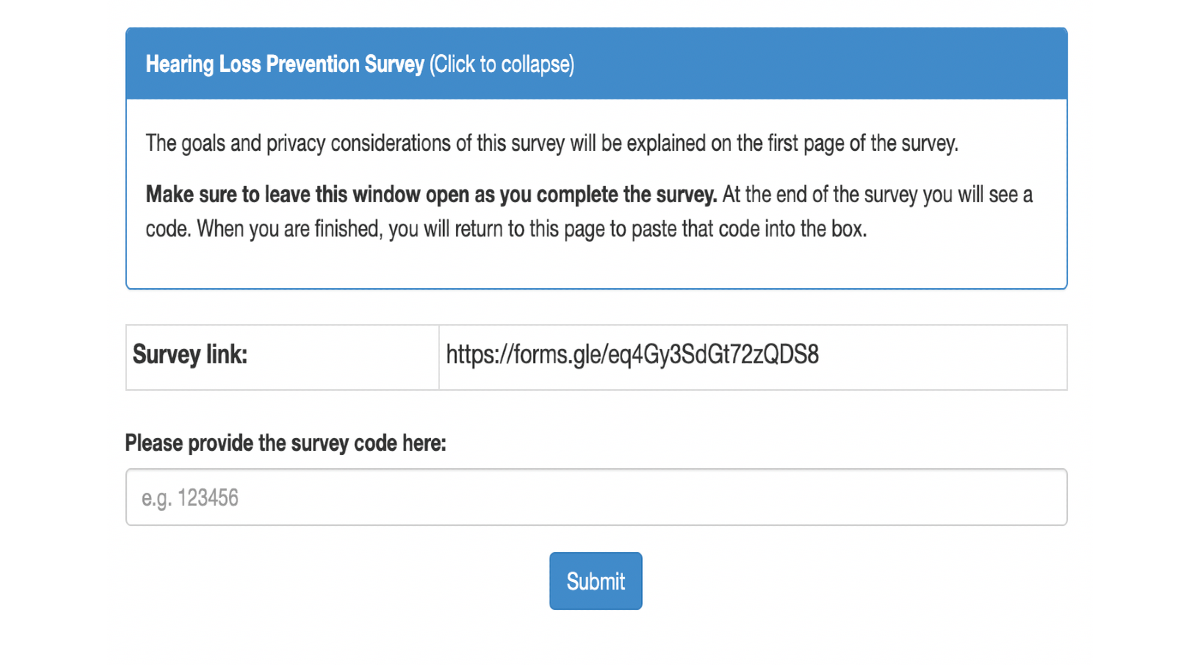
Example of a job request posted to Mechanical Turk.

An Amazon MTurk batch was created using the “survey link” template. A picture of what MTurk workers saw can be found in Figure 1. After determining the average survey completion time using a previous MTurk batch, workers were allotted 4 minutes to complete the survey and were compensated with US $0.50 each. Participants were given an estimate of the length of time of the survey in the job description posting on MTurk. The job was not mandatory and all workers chose to take the task from their end. After each batch, worker qualifications (which function like digital tags on MTurk workers) were assigned to every worker ID that had taken the survey. Workers with these qualifications were then filtered out from future survey batches to prevent workers from retaking the survey.

A total of 5 batches were run to collect a total of 195 responses, with the longest batch taking 12 hours to complete and the largest batch requiring 100 responses. MTurk batches were run from July 13, 2020, to July 15, 2020. A summary of all the collected data can be found in Multimedia Appendix 2.

Completed work was rejected if the answer to the adaptive question “How do you prevent NIHL?” was incomprehensible or otherwise unusable for analysis (eg, “yes, i over time of period t0 actively NHL [*sic*]”) or meaningless as an answer to the question (eg, “NO”). Such answers reflected a carelessness in answering the survey, which likely carried over to the responses that respondents gave for other questions. As it was otherwise impossible to accurately identify which specific questions had been answered attentively, all their responses were deemed useless for analysis.

As the adaptive question “How do you prevent NIHL?” would only appear to respondents who answered that they do actively prevent NIHL, this response rejection method could not determine the attentiveness of respondents who did not actively prevent NIHL. Thus, if this removal method was continued and the data were then used, the resulting data set would have an artificially high percentage of people who did not already prevent NIHL. However, the data set could not include data filled out by inattentive respondents because of the potential to include false data that do not represent each respondent’s true beliefs. As the data points could neither be kept nor correctly removed without introducing unfair bias into the entire data set, all MTurk data were not used for any conclusive analysis. Thus, this study cannot be applied to the general public.

### Collecting Data Using Reddit

Reddit was chosen as another site to gather data because it was the easiest and cheapest way to reach a group of users based on a common interest, in this case music. Users on the site often engage in discussions around the theme of each community; these communities are called *subreddits*. The common interest in that theme necessarily preselected participants to be those who enjoyed music to some degree. The goal was to gauge the opinions of recreational music listeners who might use the theoretical product described in this study.

**Figure 2:**
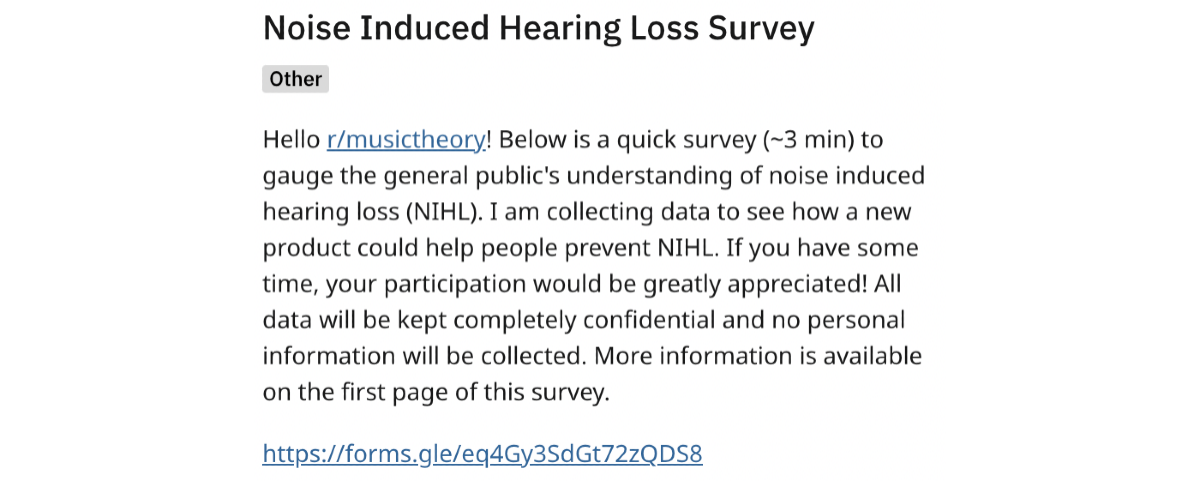
Example of a survey announcement post Reddit, as posted to the subreddit/r/musictheory.

After all MTurk batches had finished, the original survey was slightly modified to remove MTurk-specific features, such as the question about a worker ID and the survey code at the end of the survey. A Reddit post was then drafted with the survey link included, and participants were given an estimate for the survey completion time in the post’s description on Reddit. A picture of what each Reddit user saw can be found in Figure 2. The survey was not mandatory. Users self-selected to take the survey from their end and were not actively selected by researchers.

The survey was posted after confirming the approval of each subreddit’s moderators. In the end, the subreddits to which the survey was posted were /r/samplesize, /r/WeAreTheMusicMakers, /r/TameImpala, /r/indieheads, /r/musictheory, /r/deathgrips, and /r/Music. The survey remained open to responses for 56 hours starting on July 15, 2020. No incentives were provided to the respondents, financial or otherwise. We did not screen the survey data for repeat respondents, assign cookies to users, or try to determine participation rates. Similarly, IP addresses and log files were not collected or used.

### Cleaning Survey Data

As every question on the survey was mandatory in order to submit the form, completion of the survey was implicit in the survey’s submission. No survey that was terminated before completion was received. After removing the responses from MTurk workers and 3 Reddit users whose free response answers could not be used (eg, providing answers such as “Poop based fart receptacle,” “Death grips,” and “poop”), 116 responses made up the final pool of respondent data. Every response gathered from the survey can be found in the Microsoft Excel sheet located in Multimedia Appendix 2. Thus, the resulting sample was a convenience sample of music listeners from Reddit. An original, unmodified copy of the data was preserved on a spreadsheet page in the Microsoft Excel file separate from where data were manipulated to create figures.

Some assumptions were made about certain free responses to create more uniformity in the data (eg, changing the response “all ok” into being a vote for every notification type). In response to the question asking about NIHL prevention methods, 2 respondents used the additional selection choice “Other” instead of either “Exact Decibel Level” or “Estimate” to write a more detailed description of their NIHL prevention method. These responses were sorted into either the “Estimate” or “Exact” groups. The response “i do not i just try to avoid loud place [*sic*]” was counted in the “Estimate” group, and the response “a mix of the two” was counted in both the “Estimate” and “Exact” groups as half of a vote to maintain a consistent total number of respondents (n=116). After every manipulation of the data, the new set was compared with the original to ensure that all information was carried over accurately, and no information was lost or changed without reason.

### Ethical Considerations

This study is exempt from a Research Ethics Board review as the methodology falls under the criteria outlined in section 46.104 Exempt research(d)(2)(i) and (d)(2)(i)(ii) of the US Department of Health and Human Services, Basic HHS Policy for Protection of Human Research Subjects [19].

## Results

### Demographics

The following analysis stems completely from the questions used in the survey, which were quoted in the *Methods* section of this study and are included in both Multimedia Appendices 1 and 2. After removing unusable responses as detailed in the last methods section, there were a total of 116 respondents to the survey, of whom all were Reddit users. The composition of this group is as follows: 57.8% (67/116) of respondents were between the ages of 18 and 30 years, 31% (36/116) were <18 years old, 8.6% (10/116) were 31-50 years old, and 2.6% (3/116) were >50 years old.

### NIHL Awareness

Among music listeners, there was a high awareness of NIHL. Table 1 shows that 79.3% (92/116) respondents had knowledge of NIHL before taking the survey; 2% (2/90) of respondents reported being officially diagnosed with some form of NIHL, although there were a total of 4 respondents (including those 2 respondents) who described having hearing-related issues such as tinnitus and hyperacusis. This suggests that some portion of the population had undiagnosed hearing loss.

**Figure 3:**
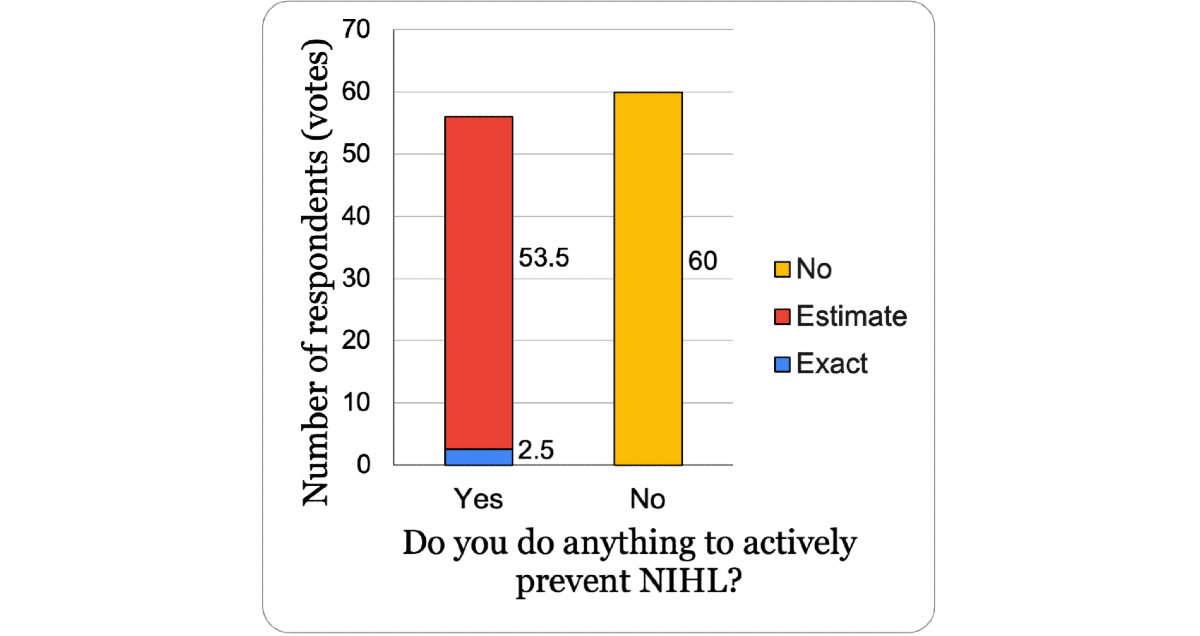
NIHL awareness and prevention methods. NIHL: noise-induced hearing loss.

Having more awareness of NIHL seemed to correlate with respondents feeling a greater risk of developing NIHL. Mapping the ordinal responses each to a value from 1 to 5 (“No Risk”=1; “Extreme Risk”=5) showed a slight increase in the mean risk that respondents felt, though an unpaired 2-tailed *t* test showed the difference was not significant (*P*=.16). As shown in Table 1, the median risk felt by all respondents was a moderate risk of developing NIHL. Regardless of prior knowledge of NIHL, about 82.8% (96/116) of all respondents felt a moderate, high, or extreme risk of developing NIHL. Although there was a high awareness of NIHL, Figure 3 shows that a majority of respondents (60/116, 51.7%) did not do anything to prevent NIHL. Of the respondents who did prevent NIHL, 96% (53.5/56) used estimates to prevent NIHL, leaving only about 4% (2.5/56) that claimed to use quantitative data to prevent NIHL.

**Table 1:** Perceived risk of developing noise-induced hearing loss (NIHL^a^; N=116).

Perceived NIHL risk	Did you have awareness of NIHL before this survey?, n (%)	
	Yes (n=92)	No (n=24)
Extreme risk	5 (5)	0 (0)
High risk	26 (28)	7 (29)
Moderate risk	48 (52)	10 (42)
Little risk	13 (14)	7 (29)
No risk	0 (0)	0 (0)

^a^NIHL: Noise-induced hearing loss.

### NIHL Risk Notification Comparisons

The NIHL risk notification type associated with the greatest likelihood of encouraging NIHL prevention was the auditory type as shown in Table 2.

The percentages shown in Table 2 represent the average likelihood of NIHL prevention across all respondents who voted for a certain NIHL risk notification type. An individual respondent’s likelihood of preventing NIHL was determined using their responses to the questions asking if they would like to receive a notification about their risk of developing NIHL, and if they would be willing to lower their media volume if reminded to. The five possible answer choices for each question were “Definitely,” “Probably,” “Maybe,” “Probably not,” and “Definitely not.” These qualitative responses were mapped to percentage values at regular 25-point intervals, starting with “Definitely not” being equivalent to 0% and ending with “Definitely” as equivalent to 100%. Each respondent’s likelihood of preventing NIHL was then estimated to be the product of their answers to the 2 questions. A table of all possible percentages and their products is presented in Table 3.

The average likelihood of eventual NIHL prevention across all respondents who voted for the auditory NIHL risk notification (n=40) was 62% (SD 24%) with a median of 56% (95% CI 50%-75%). This was significantly higher than the average across all visual NIHL risk notification voters (n=84), which was 50% (SD 28%) with a median of 50% (95% CI 38%-56%). There was no significant difference (H_1_=6.365; *P*=.19) between the auditory NIHL risk notification and the external visual NIHL risk notification, whose average probability of encouraging NIHL was 52% (SD 30%) and had a median of 57% (95% CI 38%-75%). A Kruskal-Wallis test corrected for ties revealed a significant difference (H_1_=6.848; *P*=.03) between the auditory and visual NIHL risk notifications. No significant difference was observed between visual and external visual NIHL risk notifications (H_1_=6.307; *P*=.64) either.

As shown in Table 2, where “n” reflects the number of unique respondents who chose a notification type, the NIHL risk notification that was selected the most often was the visual NIHL risk notification with 84 votes. The auditory and external visual NIHL risk notifications each received 40 votes. A total of 12 respondents chose the “None” selection, with the average chance of preventing NIHL in that group being 14% (SD 21%). A total of 3 respondents chose “Other,” and their mean likelihood of preventing NIHL was 21% (SD 16%). The three “Other” responses provided by respondents are listed below exactly as they were typed by each respondent:

1. “Nonintrusive pop-up in a top empty corner, warning for SERIOUS issues (that can be deactivated) and not bug you about everything, as phones do.”
2. “Vibration maybe?”
3. “Haptic, on a watch or something.”

**Table 2:** Average likelihood of each noise-induced hearing loss (NIHL^a^) risk notification type to encourage NIHL prevention.

NIHL risk notification type	Likelihood (%), mean (SD)
Auditory (n=40)	62 (24)
External visual (n=40)	52 (29)
Visual (n=84)	50 (28)
Other (n=3)	21 (16)
None (n=12)	14 (21)

^a^NIHL: Noise-induced hearing loss.

**Table 3:** Response ranking system.

Possible responses	Definitely (%)	Probably (%)	Maybe (%)	Probably not (%)	Definitely not (%)
Definitely	100	75	50	25	0
Probably	75	56.3	38.5	18.8	0
Maybe	50	38.5	25	12.5	0
Probably not	25	18.8	12.5	6.3	0
Definitely not	0	0	0	0	0

### Respondent Willingness to Prevent NIHL

As shown in Table 4, on average, most respondents were inclined to lower the volume of audio entering their ears. The percentages listed in Table 4 are related to the total number of respondents for that age range. Regardless of age, 66.4% (77/116) of respondents would either “Definitely” or “Probably” turn down their audio volume if reminded to. In addition, of those respondents who did not already prevent NIHL, 57% (34/60) would either “Definitely” or “Probably” turn down their audio volume. A total of 18.9% (22/116) respondents chose “Maybe,” and 14.7% (17/116) respondents chose either “Probably not” or “Definitely not.”

**Table 4:** Table of respondent willingness to turn down audio by age group.

Respondents	Would you lower the volume of audio entering your ear if you were reminded to?, n (%)				
	Definitely	Probably	Maybe	Probably not	Definitely not
<18 years old (n=36)	10 (27.8)	13 (36.1)	9 (25)	3 (8.3)	1 (2.8)
18-30 years old (n=67)	22 (32.8)	23 (34.3)	12 (17.9)	10 (14.9)	0 (0)
31-50 years old (n=10)	4 (40)	3 (30)	1 (10)	1 (10)	1 (10)
>51 years old (n=3)	0 (0)	2 (66.7)	0 (0)	1 (33.3)	0 (0)
All respondents (n=116)	36 (31)	41 (35.3)	22 (19)	15 (12.9)	2 (1.7)

## Discussion

### Principal Findings

Overall, the data seemed to suggest that technology that can provide music listeners with a real-time assessment of their risk of developing NIHL would be useful for encouraging NIHL prevention. A majority (60/116, 51.7%) of the population did not practice any form of NIHL prevention despite 79.3% (92/116) of respondents having some awareness of NIHL. Even within the population that did prevent NIHL, only about 4% (2.5/56) reported using noise level data to aid in the prevention of NIHL. Across the entire population of respondents, 82.8% (96/116) felt more than a little risk of developing NIHL. None of the respondents reported a complete lack of risk. Of the respondents, 66.3% (77/116) were willing to lower their audio volume if they were reminded to, and the NIHL risk notification that had the highest mean estimated chance of encouraging NIHL prevention was the auditory NIHL risk notification at 61.9%. The NIHL risk notification type respondents preferred the most seemed to be the visual NIHL risk notification type with 84 total votes.

As there was a high percentage (53.5/56, 96%) of respondents who used estimates in their prevention of NIHL, and 51.7% (60/116) of all respondents did not practice preventing NIHL, technology that can both encourage NIHL prevention in those who do not already prevent it and provide more accurate data to those who currently estimate their noise exposure would be useful. Without data to guide prevention methods, consumers of music may either not truly prevent NIHL or be listening to music at too low a volume to enjoy the activity. For those who do not already prevent NIHL, technology that encourages NIHL prevention could allow them to enjoy their music for a longer period of their lives. Even though a majority of the population did have awareness of NIHL, technology that informs the user of their risk of developing NIHL could still be useful because most respondents did not prevent it, do not have accurate methods of actively analyzing and preventing NIHL on their own, and are willing to reduce their music volume if reminded to.

### Future Work

The results of this study readily lend themselves to many opportunities for future research and overall seem to suggest an encouraging environment for the future of NIHL awareness and prevention. Instead of increasing NIHL awareness through conventional methods such as bolstering NIHL education in schools, future efforts to increase NIHL awareness and prevention could take the form of more targeted individual encouragement through technology. Below are various flaws in the research methodology that seriously affected the conclusions that could be drawn, which future studies would do well to improve upon.

### Study Population

In choosing Reddit communities already related to music, the survey collection method likely encouraged a bias toward respondents who heavily listened to music, and thus, may disproportionately favor using an NIHL risk notification compared with the general public. This study may also not have fully sampled a group that was representative of people who regularly listen to music, as those music listeners who were not in the chosen subreddits were not given the same opportunity to supply their responses. This bias may have resulted in a seemingly greater concern for preserving hearing ability than may truly reflect the population of people who would benefit from awareness about NIHL. In addition, without an external incentive to complete the survey, respondents who did go out of their way to supply responses might have already believed more in the utility of an NIHL risk notification. To obtain a more accurate estimate of the general public’s perception of NIHL risk notifications, future surveys should use a population more representative of the general public and provide an additional incentive to complete the survey, such as a monetary reward. A more randomized sampling method should also be used to create a sample of those who enjoy music. Future surveys should also try to include more participants aged ≥31 years, as well as possibly control for the countries of each participant. The timing of the survey’s posting may also be important to for control in future studies.

### Data Collection

A more quantitative data collection method to determine the usefulness of each NIHL risk notification should be used in the future. The Kruskal-Wallis test used here inflated the possibility of type 1 errors, especially with having to correct for ties in the data. Future studies could also incorporate data from pure tone audiometry measurements and otoacoustic emission tests. There should also be a way to assess the true level of NIHL knowledge each participant had, instead of letting them self-report either a “Yes” or a “No.” It may also be easier to gauge how respondents feel toward individual NIHL risk notification types by having a more direct rating system for each individual NIHL risk notification. A picture of each hypothetical NIHL risk notification would also have made each notification type easier to conceptualize for respondents.

### MTurk Data

Although the MTurk data may not truly reflect a more general population of people, some apparent differences between the Reddit and MTurk data suggest that NIHL risk notifications might not be as useful for the general public. A much greater percentage (35/63, 56%) of MTurk workers than Reddit users (2.5/56, 4.5%) indicated that they used exact decibel levels to inform their NIHL prevention. This seems counterintuitive because conventional logic might suggest that people who interact with audio more would be more likely to have the technology and inclination to accurately track their risk. In addition, a higher percentage of MTurk respondents (54/121, 44.6%) than Reddit respondents (12/116, 10.3%) indicated that none of the listed notification types were good, suggesting that a different notification type may be necessary to encourage NIHL prevention in the general public. Interestingly, a much higher percentage (55/121, 45.5%) of MTurk respondents indicated that they had officially diagnosed NIHL compared with 1.7% (2/116) of respondents in the Reddit population. It should be noted again that the MTurk data can only indicate a need to more accurately study the general public to either confirm or reject these trends, as the various flaws in the response collection process delegitimize the significance of any conclusions that could be drawn. Many MTurk responses also suggested that respondents did not understand English very well, which may have led to them unintentionally answering questions inaccurately.

### Changing Behaviors

Despite this survey describing some likelihood of NIHL risk notifications (especially the auditory type) successfully encouraging NIHL prevention, significantly more research needs to be done to determine which NIHL risk notification could actually encourage lasting behavioral change in practice. Within each of the three NIHL risk notification types identified here, there are numerous design choices to be considered during the production of actual prototypes, which may change the overall effectiveness of each NIHL risk notification. Choices such as using more images over text in a visual notification or perhaps using different people’s voices for the audio NIHL risk notification all may need to be considered. Similar to the work by Kaplan-Neeman et al [18], future work must be done with all forms of NIHL risk notifications to conclusively determine what system and data presentation methods might actually encourage user change.

### Moving Forward

**Figure 4:**
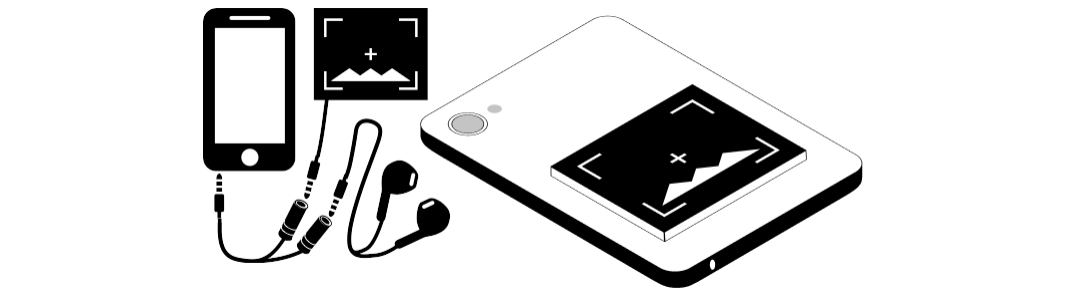
A visual screen is attached to the back of a mobile phone, similar to existing portable digital-to-analog converters. The audio connection is facilitated by a male to 2-female audio splitter cable.

The three NIHL risk notifications described here correlate to 3 distinct types of consumer products that could be created in the future. External visual NIHL risk notifications could take the form of wearable watches, as mentioned earlier, or something similar to Figure 4, where a physical connection to the user’s media source is maintained. “Audio Jack” and “screen” icon sources from the study by Daksina [20] and Ahmad [21], respectively. The overall figure was designed by author DTZ. It should be noted that a potential flaw in the utility of this risk notification type may be the current consumer trend toward wireless listening equipment. The audio NIHL risk notification was conceptualized as a software and hardware package that both performed the required noise exposure calculations and provided the NIHL risk notifications as audio cues all from within the user’s personal listening equipment. The visual NIHL risk notification was mainly envisioned as an app on the user’s phone to measure the audio equipment output and then visually display NIHL warnings to the user.

Another NIHL risk notification method was later theorized by Tim Nieuwenhuis, which adds to the potential utility of the visual notification method. In the idea, an external physical device would act as the user’s ear and measure the noise output of the personal listening device as the user plays a provided audio file for calibration. The device would then send the data back to the user’s phone. The NIHL risk notification app combines the output data with the audio signal data going out of the phone to build a model for estimating the noise that enters the user’s ear. The notification itself would likely still appear on the app. There is potential for this method to be applicable to recreational music listening through speakers, although an issue then might be determining the user’s position in a room.

It may also be worthwhile to adapt NIHL risk notifications for contexts beyond recreational music listening at home. Future NIHL risk notifications could be created for other situations with a high risk of causing NIHL, such as playing an instrument or attending music festivals [6,22]. A version might be created to augment the usefulness of existing earplugs. Significant consideration should be given to the fact that the NIHL risk notifications theorized here rely heavily on being able to use the user’s personal listening equipment to calculate a time-weighted dBA average of noise exposure entering the ear, which may not be as feasible in other contexts or within equipment such as earplugs.

## Appendix

Multimedia Appendix 1 The complete Google Form as presented to survey respondents.

Multimedia Appendix 2 Complete catalog of all survey data collected in this study. The compiled data from every survey batch are on the spreadsheet page titled “Compiled,” which has already been sorted. The “Cleaning Data” page was where all the data were manipulated to create graphs. Preliminary data tables can be found on the far-right side of the page.

## Abbreviations

MTurk: Mechanical Turk
NIHL: noise-induced hearing loss
NIOSH: National Institute for Occupational Safety and Health
