# Supplementary material for: Encouraging Hearing Loss Prevention in Music Listeners Using Personalized Technology: Questionnaire Study
Source: JMIR Form Res. 2022 Jun 27;6(6):e24903. doi: 10.2196/24903 (PMC9274393; doi:10.2196/24903)
Supplement: Multimedia Appendix 1 [file formative_v6i6e24903_app1.pdf]

Hearing Loss Survey

Most of the sounds we hear are at safe levels, but when sounds are too loud or if you are exposed to loud sounds over a period of time, they can be harmful to the ear and cause Noise Induced Hearing Loss (NIHL).

Even if you can't tell that you are damaging your hearing, you could have trouble hearing in the future, such as not being able to understand other people when they talk, especially on the phone or in a noisy room. [US National Institute on Deafness and Other Communication Disorders]

An estimated 12.5% of children and adolescents aged 6–19 years (approximately 5.2 million in the US) and 17% of adults aged 20–69 years (approximately 26 million in the US) have NIHL. [US Center for Disease Control and Prevention]

The purpose of this survey is to understand how technology might used to help people prevent NIHL.

Participation in this study is on a purely voluntary basis. We do not collect your email or any other identifiable information. You will be asked to complete a brief survey. The results from this study may be published or presented. Your involvement in this study is appreciated, but you may quit participation at any time if you would like.

If you have questions about this project, please contact blorenge50@yahoo.com or write them in the space provided at the very end of this survey.

* Required

If you have read and understand the above statements, please click the button below to indicate your consent to participate in this study. *

- ○ Yes, I wish to participate in this survey.
- ○ No, I decline the opportunity to participate in this survey. Your data will be deleted from the collected responses.

What is your current age? *

- Under 18
- 18-30
- 31-50
- 51+

Before this survey, had you heard of Noise Induced Hearing Loss (NIHL)? *

- Yes
- No

Do you believe you are at risk for developing NIHL? *

- No Risk
- Little Risk
- Moderate Risk
- High Risk
- Extreme Risk

Have you been diagnosed with NIHL? *

- Yes
- No

Next

Never submit passwords through Google Forms.

This form was created inside of Blair Academy. Report Abuse

Google Forms

Hearing Loss Survey

* Required

Cause of NIHL

What do you believe was the cause of your hearing loss? *

- ◯ Congenital Problem
- ◯ Loud Sounds Over Time
- ◯ A Single Loud Noise
- ◯ Age Related
- ◯ Don't Know
- ◯ Other:

Page 2 of 5

Back Next

Never submit passwords through Google Forms.

This form was created inside of Blair Academy. Report Abuse

Google Forms

Hearing Loss Survey

* Required

NIHL Prevention

Do you do anything to actively prevent NIHL? *

- Yes
- No

Page 3 of 5

Back Next

Never submit passwords through Google Forms.

This form was created inside of Blair Academy. Report Abuse

Google Forms

Hearing Loss Survey

* Required

Prevention Method

Do you estimate an appropriate loudness based on personal preference or do you track the exact decibel level of every sound that enters your ear? *

- Estimate
- Exact Decibel Level
- Other:

What do you do to actively prevent NIHL? *

Your answer

Back Next

Never submit passwords through Google Forms.

This form was created inside of Blair Academy. Report Abuse

Google Forms

Hearing Loss Survey

* Required

Hearing Loss Notifications

Would you like to receive some notification about your risk of developing NIHL from music or the environment around you? *

- Definitely
- Probably
- Maybe
- Probably Not
- Definitely Not

Would you lower the volume of audio entering your ear if you were reminded to? *

- Definitely
- Probably
- Maybe
- Probably Not
- Definitely Not

Please select all of the notification methods that you would prefer to receive. If you would prefer some other notification method, please write it in the "Other" option. If you prefer none of the methods please only select "None". *

- [ ] Audio Notifications (e.g. a message through your earbuds)
- [ ] External Visual Notifications (e.g. a display on your phone case)
- [ ] Visual Notifications On Your Phone (e.g. on an app or the phone's lock screen).
- [ ] None
- [ ] Other:

If there is anything else you would like to share about NIHL or how technology might be used to help prevent NIHL, please do so here.

Your answer

Back Submit

Never submit passwords through Google Forms.

This form was created inside of Blair Academy. Report Abuse

Google Forms
